# A simple and effective deep neural network based QRS complex detection method on ECG signal

**DOI:** 10.3389/fphys.2024.1384356

**Published:** 2024-07-15

**Authors:** Wei Zhao, Zhenqi Li, Jing Hu, Yunju Ma

**Affiliations:** Central Research Institute, Guangzhou Shiyuan Electronics Co., Ltd., Guangzhou, China

**Keywords:** electrocardiogram (ECG), QRS complex, Qrs complex detection, deep learning, QRS complex boundary

## Abstract

**Introduction:** The QRS complex is the most prominent waveform within the electrocardiograph (ECG) signal. The accurate detection of the QRS complex is an essential step in the ECG analysis algorithm, which can provide fundamental information for the monitoring and diagnosis of the cardiovascular diseases.

**Methods:** Seven public ECG datasets were used in the experiments. A simple and effective QRS complex detection algorithm based on the deep neural network (DNN) was proposed. The DNN model was composed of two parts: a feature pyramid network (FPN) based backbone with dual input channels to generate the feature maps, and a location head to predict the probability of point belonging to the QRS complex. The depthwise convolution was applied to reduce the parameters of the DNN model. Furthermore, a novel training strategy was developed. The target of the DNN model was generated by using the points within 75 milliseconds and beyond 150 milliseconds from the closest annotated QRS complexes, and artificial simulated ECG segments with high heart rates were generated in the data augmentation. The number of parameters and floating point operations (FLOPs) of our model was 26976 and 9.90M, respectively.

**Results:** The proposed method was evaluated through a cross-dataset test and compared with the sophisticated state-of-the-art methods. On the MITBIH NST, the proposed method demonstrated slightly better sensitivity (95.59% vs. 95.55%) and lower presicion (91.03% vs. 92.93%). On the CPSC 2019, the proposed method have similar sensitivity (95.15% vs.95.13%) and better precision (91.75% vs. 82.03%).

**Discussion:** Experimental results show the proposed algorithm achieved a comparable performance with only a few parameters and FLOPs, which would be useful for the application of ECG analysis on the wearable device.

## 1 Introduction

The electrocardiograph (ECG) represents the electrical activity of heartbeat. It is a widely-used tool for examining the cardiovascular diseases (CVDs) due to the characteristic of low-cost and painless. A typical ECG waveform of heartbeat is composed of several characteristic waveforms, such as the P wave, QRS complex and T wave. It is thought that the amplitude, duration, contour and number of the waveforms, and the interval between the peak of waveforms provide fundamental information for the monitoring and diagnosis of CVDs (Taylor, 2008). Because the QRS complex is the most prominent waveform, accurate detection of the QRS complex is essential in the ECG analysis algorithms (Kohler et al., 2002).

Many algorithms for the QRS complex detection have been proposed since the last several decades. Most of detectors can be divided into two fundamental parts, the preprocessing and the identification (Kohler et al., 2002). In the preprocessing stage, the QRS complex was enhanced and the noise was attenuated. In the identification stage, the QRS complex was determined and the false posive were removed. For example, in the P&T method (Pan and Tompkins, 1985), one of the most popular algorithms, after bandpass filtering of the ECG signal, the QRS complex was highlighted via the procedure of five-point derivative, squaring and moving window integration. Eventually the QRS complex was determined by adaptively thresholding on the preprocessed signal and filtered ECG signal. Hamilton *et al*. (Hamilton and Tompkins, 1986) slightly modified the preprocessing technique of P&T’s method, which determine the QRS complex by more complex rules. It is reported (Liu F. et al., 2018) that although the traditional signal processing based methods can accurately locate the QRS complex on the noise-free ECG signal, the performance of such algorithms significantly decreases on the ECG segments with severe artifacts.

Recently, the deep learning (DL) techniques have been widely used to improve the performance of QRS comple detection. The powerful representation ability of the DL model, combined with the end-to-end training process enable it to more effectively enhance and identify the QRS complex. In the work (Zahid et al., 2022) the detection of QRS complex was formulated as a 1D segmentation task, and the U-Net, a popular medical image segmention approach, was used to enhance the QRS complex. The model produce a high response at the location of QRS complex, and a low response elsewhere. The QRS complex were determined by a given threshold. Furthermore, considering that the bidirectional Long Short Term Memory (BLSTM) can extract features during a long period, in work (He et al., 2021), the QRS complex were enhanced by the DNN model by combining the U-net with BLSTM.

It is reported that the size of receptive field is a crucial issue in the visual tasks, and enlarging the receptive field can improve the accuracy of the location method (Liu Y. et al., 2018). The work (Lee et al., 2019) demonstrated the DL model with a sufficiently large-sized receptive field that can cover adjacent heartbeats achieved good performance on the capacity ECG signal. A similar sized receptive field would also be required for the detectors applied on the ECG signal. However, most traditional CNN based QRS complex detectors were derived from image processing techniques, and adopted small-sized kernel (about 3 or 5 samples). Although several convolutional layers and pooling layers were used to build the model, the receptive field of model was still relatively small. The LSTM-based method can extract features over a long period, but it has a large number of parameters, which would be disadvantageous for the generalization ability of detector.

On the other hand, although the accurate identification of the endpoints of the object is critical in the segmentation task, it would be unnecessary for the QRS complex detection, especially in the training stage. Since most ECG datasets lack annotations of QRS complex fiducial points, a common approach for generating the target vector is to define the QRS complex region with a fixed range around the location of annotated QRS complex, where the samples within the region are labeled as positive and those outside the region as negative. However, the durations of QRS complex vary among different individuals, especially for patients with CVDs. Therefore, the fixed range is difficult to precisely delineate the QRS complex. This leads to assigning labels to adjacent samples with similar presence, which could degrade the performance of the detector.

In order to address the above problems, a simple QRS detection method was developed and an innovative training strategy was designed in the work. The DNN model is based on fully convolutional network (FCN) architecture, without the application of sophisticated modules such as LSTM. The model has a kernel size of 19 samples and operates at a sample rate of 250 Hz. This allows the model to have a maximal receptive field of approximately 2 s, which covers the longest interval between normal heartbeats (Taylor, 2008). Additionally, considering that the joint analysis of multi-lead signal will provide better robustness in the presence of noise in any single lead, the model is designed to have dual input channels. When the model was operated on the ECG signal with single lead, the dat in the second channel of the model is replicated of the data from the first channel. In the training, the points on the boundary of the QRS complex were excluded to generate the target vector for the DNN model. Besides, due to the limited availability of high heart rate segments in the existing datasets, the artificially simulated ECG segments of fast heart rates generated in the data augmentation, Seven public ECG datasets were used to evaluate the proposed methods, and experimental results show that the proposed method achieved a good performance on the cross-dataset test, making it useful for wearable ECG devices. The main contributions of this work were as follows: 1. A FCN based model with a large-sized kernel, allowing for a maximal receptive field of approximately 2 s 2. Incorporation of dual-lead ECG signal analysis. 3. Elimination of the border points of the QRS complex in the generation of the target vector. 4. Inclusion of artificially simulated ECG segments with fast heart rates in the data augmentation.

The rest of this paper is organized as follows. Section 2 introduced the recent QRS complex detection method. Section 3 presents the proposed QRS complex detection method. Section 4 gives the experimental results of developed method and several existing state-of-the-art detectors. Section 5 discusses the method. And Section 6 concludes the work.

## 2 Recent work

The enhancement and determination of the QRS complex are the two primary stages in QRS complex detection, and several advancements have been made in these approaches. For the traditional signal processing-based detectors, the QRS complex was enhanced by using linear or non-linear filters and determined based on sophisticated decision rules. In the work (Khamis et al., 2016), the feature signal of the QRS complex was generated by multiplying the signal derivative and amplitude envelope, and the threshold was calculated using a morphological closing operation with maximum and minimum filters. A signal quality mask was also utilized to eliminate false positives. In the study (Orlandic et al., 2019), an enhancement filter based on relative-energy, which is the ratio between the energies of a long sliding window and a short sliding window, was designed, and the QRS complex was identified using a hysteresis comparator with two adaptive thresholds. In the work (Rodrigues et al., 2021), a double derivative-based pre-processing method was employed to enhance the QRS complex, and the thresholds were determined using the finite state machine approach. In the study (De Giovanni et al., 2023), a Bayesian filter integrated with clustering techniques was applied to compute the expected position of the QRS complex.

Due to the power representation of deep learning model, the deep learning technique was applied to detect the QRS complex. Some researchers have designed detector based on ECG segments. In these study, the ECG signals are divided into several segments of fixed length, which are then fed to a deep model. In the publication (Šarlija et al., 2017), the ECG signal was splitting into segments of 400 milliseconds. The model consisted of two convolutional layer and two fully connected layers. Similarly, in the publication (Wang and Zou, 2019) the ECG signal was also divided into 400 milliseconds segments. The model utilized two parallel residual networks with kernel sizes of 7 samples and 3 samples respectively. In the work (Xiang et al., 2018), the ECG signal was segmented into slices of 56 samples. After averaging and difference operation, a two-level convolutional neural network (CNN) was designed to learn the features at the part-level and object-level of the QRS complex, The resulting features were concatenated and passed through a multi-layer perceptron (MLP) for the classification. The slice-based method can capture the local characteristic of QRS complex. However, the contextual information of segments is lost for these approaches.

In order to further improve the performance, the utilization of medical image segmentation technique and LSTM-based approaches has become increasingly popular. In the work (Zahid et al., 2022) The U-Net was employed to segment the QRS complex. Cai *et al.* (Cai and Hu, 2020) utilized the BLSTM after the multiple parallel dilated convolutional blocks to predict the QRS complex. In the work (He et al., 2021), The DNN model enhanced the QRS complex by combining the U-Net with BLSTM.

## 3 Materials and methods

Seven public datasets were used in the experiments, five of which from the PhysioNet (Goldberger et al., 2000): MIT-BIH Arrhythmia (Moody and Mark, 2001) (AR), MIT-BIH Supraventricular Arrhythmia Database (SV) (Greenwald, 1990), MIT-BIH Noise Stress Test (GB et al., 1984) (NST), MIT-BIH ST Change (STC) (P, 1983) and European ST-T (ST-T) (Taddei et al., 1992). Additionally, the High Intensity Exercise (HIE) (De Giovanni et al., 2023) and the dataset of 2nd China Physiological Signal Challenge (CPSC 2019) (Hongxiang et al., 2019) were include. For the AR dataset, 44 non-pace records were separated into two subsets (AR_DS1 and AR_DS2), according to the division scheme proposed by the work (de Chazal et al., 2004). The NST dataset was created by adding calibrated amplitude of noise on the two clean records (118 and 119) from the AR dataset, with the SNRs of −6 db, 0 db, 6 db, 12 db, 18 db and 24 db for the noisy segments. Record 315 in STC and Record e0204 in ST-T were not used in the experiment. The information of these datasets was summarized in Table 1.

**TABLE 1:** The datasets used in experiments.

Dataset	Record	Beat	Lead	Length	Sampling rate (Hz)
AR_DS1 (19)	22	51021	2	30 min	360
AR_DS2 (19)	22	49712	2	30 min	360
NST (21)	12	25590	2	30 min	360
SV(20)	78	184583	2	30 min	128
CPSC 2019 (24)	2000	29447	1	10 s	500
ST-T (Taddei et al., 1992)	89	783244	2	2 h	250
STC (P, 1983)	27	73192	1–2	784–4032 s	360
HIE (De Giovanni et al., 2023)	20	5691	1	80–100 s	250

**FIGURE 1:**
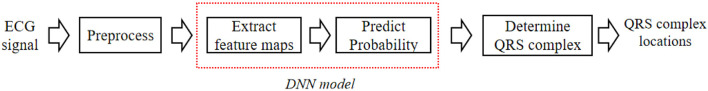
The flowchart of proposed method.

The flowchart of our method is illustrated in Figure 1. First, the raw ECG signal was preprocessed. Then, the signal was fed into the DNN model and the heat maps were generated, which indicated the probability of each point belonging to the QRS complex. Finally, QRS complexes were determined based on the heat maps, and the locations of the predicted QRS complex were saved.

**FIGURE 2:**
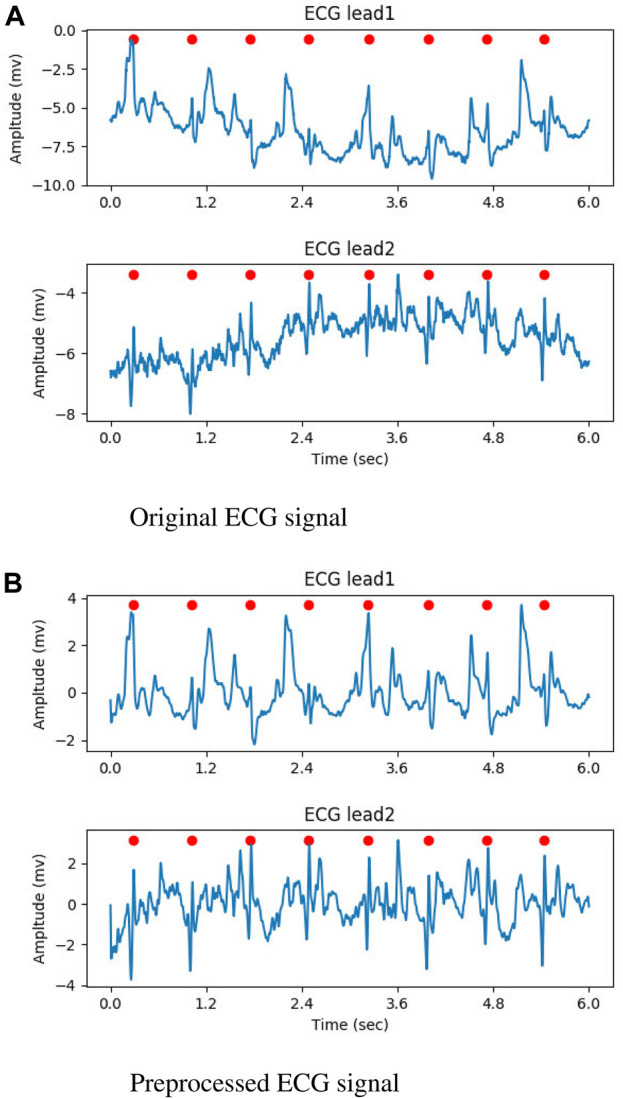
The result of preprocessed, the QRS complexes were marked by red circles. **(A)** Original ECG signal. **(B)** Preprocessed ECG signal.

In the preprocessing stage, the input ECG signal was resampled to 250Hz, and the noise was attenuated using a butterworth filter with a band-pass range of 0.5–35 Hz. At last, Z-score normalization was performed on each lead. For the raw ECG signal with a single lead, the second input channel of the DNN model was created by duplicating the preprocessed signal. Figures 2A, B illustrate the original ECG signal and the preprocessed result, respectively. In both figures, the QRS complexes are indicated by the red circles.

**FIGURE 3:**
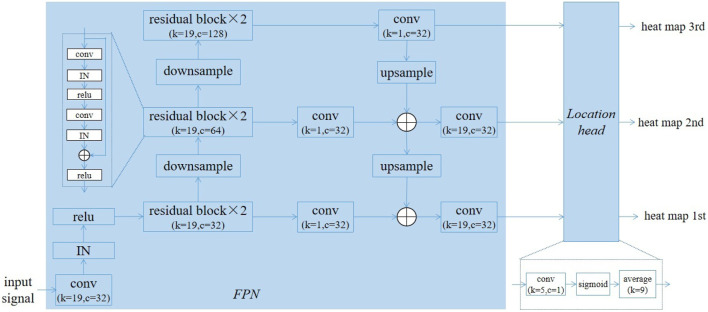
The architecture of proposed deep learning model.

The proposed DNN model was composed of two modules, a feature pyramid network (FPN) (Lin et al., 2017)-based backbone for extracting features maps at three resolutions, and a location head for predicting the probability of each points belonging to the QRS complex. Figure 3 gives the structure of our model. In the FPN, there were six residual blocks and six convolutional layers. The convolutional layers following the residual blocks were used to adjust the channels, so their kernel size were 1 (k = 1), while the kernel size for other convolutional layers was 19 (k = 19). The number of channels in the residual blocks were 32 (c = 32), 64 (c = 64), and 128 (c = 128). The instance normalization (IN) and linear rectification function (relu) were used as the normalization and activation function, respectively. Downsampling was performed using convolution with a kernel size of 1 and stride of 2, and upsampling was achieved through the nearest neighbour interporation. In order to reduce the number of parameters, depthwise convolution was employed in the convolutional layers with the kernel size of 19 except the first one.

**FIGURE 4:**
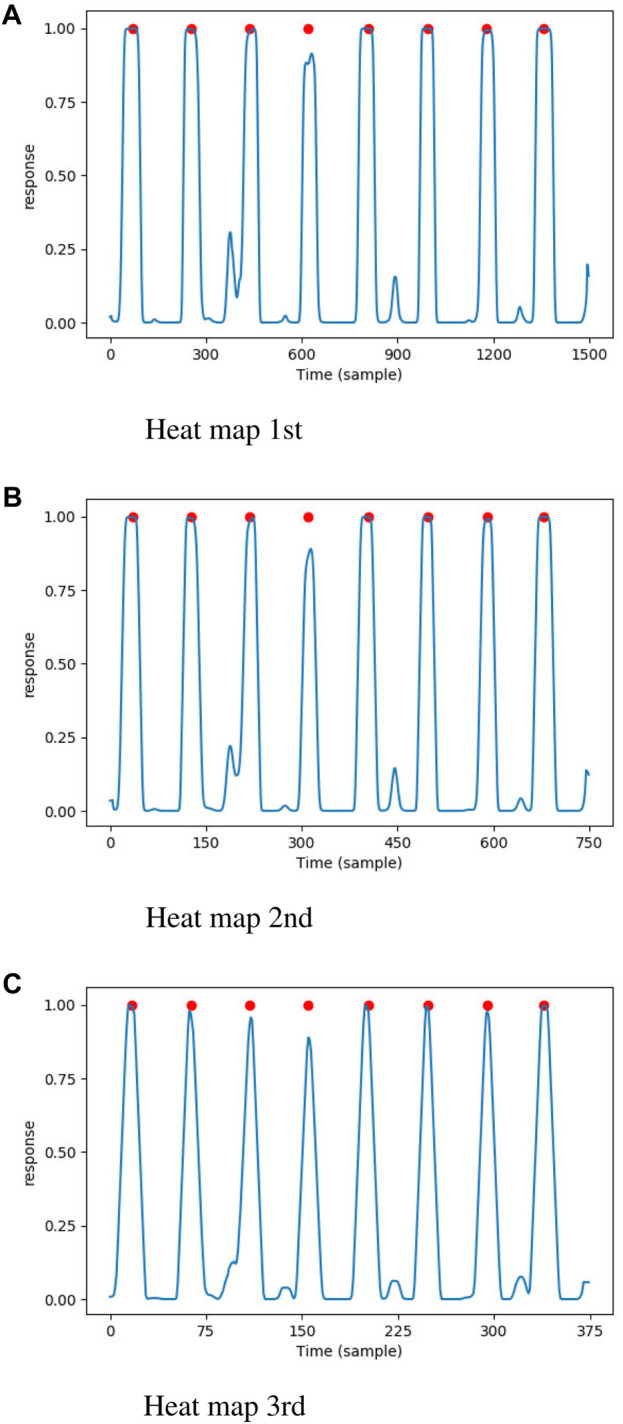
The heat maps generated by proposed DNN model, the annotated QRS complexes were marked by red circle. **(A)** Heat map 1st. **(B)** Heat map 2nd. **(C)** Heat map 3rd.

The location head consisted of a convolutional layer with kernel size of 5 and a single channel, a sigmoid layer and an average pooling layer with size of 9. Let the size of input ECG signal be l×2 , where l represents the length. The length of three heat maps were l , (l/2) and (l/4) respectively. The three heat maps are shown in Figures 4A–C, where the QRS complexes are indicated by the red circles. The DNN model generated a high response (close to 1.0) for the points near the QRS complexes. In total, the number of parameters and the floating point operations (FLOPs) of proposed model was 26976 and 9.90 million respectively.

**FIGURE 5:**
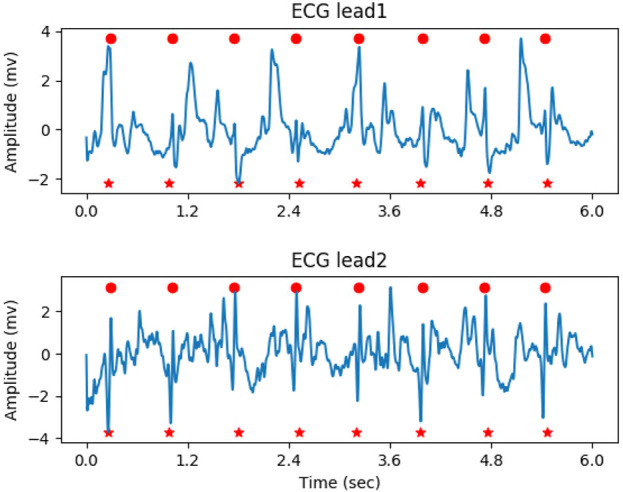
The result of QRS complex detection on ECG signal, the predicted and annotated QRS complex were marked by the red vertical dashed line and red circle respectively.

In the determination of the QRS complex, local maximal points with a response greater than the given threshold (0.5 in the experiment) were initially considered as the location of QRS complex candidates. Next, the process of non-maximal suppression (NMS) was performed to remove the false positives, as follows: 1. A check-list was generated by sorting the candidates in descending order based on their probability. 2. The candidate with the highest probability in the check-list was marked as the true positive, and its location was saved. 3. Other candidates close to the current candidate within the threshold (200 milliseconds in the experiment) were marked as false positives. 4. The true positive and false positives were removed from the check-list. 5. Steps 2-4 were repeated until the check-list was empty. Figure 5 illustrates the result of QRS complex detection on the ECG signal. The annotated QRS complexes and the predicted results were marked with red circles and red dashed vertical lines, respectively. The proposed method can accurately detect the QRS complex on the noisy ECG segment.

**FIGURE 6:**
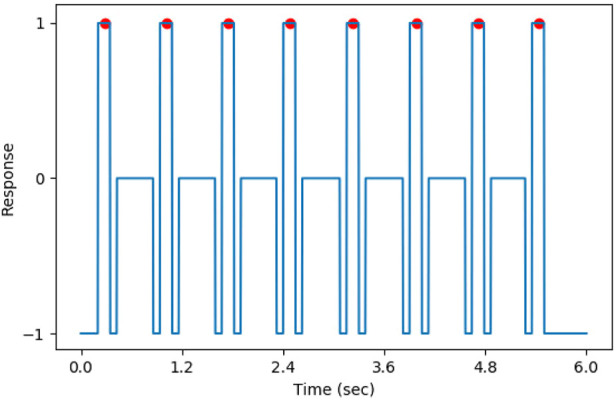
The target of DNN model, the QRS complexes were marked by the red circle.

In the training, the targets of the DNN model were created by relabeling the annotations on vectors with the same size as the heat maps. For points between the first QRS complex and the last QRS complex on the target vectors, if the distance to the closest QRS complex was less than 75 milliseconds, its value was set to 1 (positive). Otherwise, if the interval was more than 150 milliseconds, the value of the point was set to 0 (negative). The points that were neither positive nor negative were set to −1. Additionally, in order to avoid the influence of incomplete QRS complexes on training, for the points located in the initially 200 milliseconds and the last 200 milliseconds, their values were also set to −1. The points whose value was −1 were ignored when calculating the loss. The target of the DNN model is shown in Figure 6, where the QRS complexes are indicated by red circles.

**FIGURE 7:**
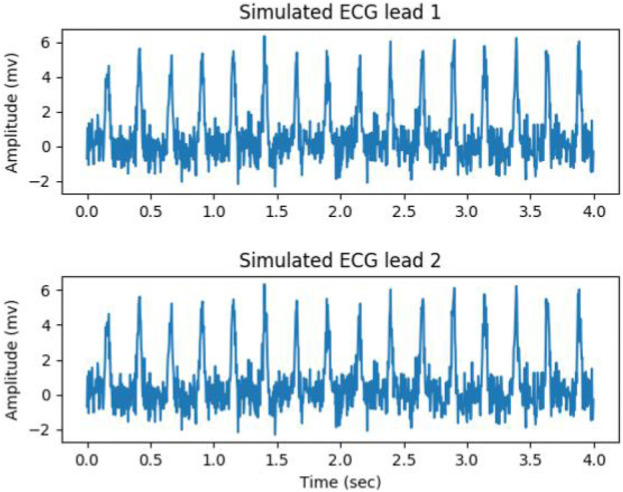
Simulated ECG segment with a bpm of 240 and SNR of 6 db, the amplitude and duration of the QRS complex were 5 mV and 80 millisecond.

The data augmention was performed from the three aspects. Firstly, artificially simulated ECG signals were generated, with BPM (beats per minute) ranging from 200 to 280. The range of amplitude and duration of the QRS complex was from 3 to 5 mV and from 50 to 100 milliseconds, respectively. The white noise with a signal-to-noise ratio (SNR) ranging from 4 db to 12 db were mixed with the generated ECG signals. Figure 7 gives a simulated ECG segment with a BPM of 240 and SNR of 6 db. The amplitude and duration of the QRS complex were 5 mV and 80 millisecond, respectively. Secondly, the ECG signal was mixed with three types of noise: baseline wandering (BW), muscle artifact (MA) and electrode motion artifact (EM). The sinusoidal function was used as BW, with its frequency determined by random sampling from three Gaussian distributions. The centers of the Gaussian distribution were 0.05, 0.15 and 0.25, respectively, and the standard deviations were one-third of center. The white noise was used as MA, and the EM was created by filtering the MA with a band-pass filter ranging from 5 Hz to 15 Hz. Thirdly, the signal from any channel was flipping, and the order of the channels in the signal was changed.

## 4 Experimental results

The proposed DNN model was developed using the PyTorch (Paszke et al., 2019), a popular deep learning library. The BCE (binary cross entropy) was calculated as the loss function, and the Adam optimizer was selected with a learning rate of 1e-3. The model was trained for 5 epochs using a batch size of 32 and weight decay of 1e-8, and the model that achieved the lowest error on the validation data was saved as the best model and used for test. The algorithm was implemented on the workstation with an Intel Xeon CPU E5-2650 and a GeForce GTX 1080 Ti.

In the experiments, our algorithm was trained and validated on the AR_DS2 dataset and tested on other datasets. The records used for the training and validation were 100, 103, 105, 111, 113, 117, 121, 123, 200, 202, 210, 212, 213, 214, 219 and 221, 222, 228, 231, 232, 233, 234, respectively. The signals from the training and validation data were sliced into segments with a length of 60 s, and the signals from test data was directly fed to the algorithm without being segmented.

According to the EC57 standard (AAMI, 2012), the beats can be categorized into five types: N (beat that does not fall into the types of S, V, F or Q), S (supraventricular ectopic beat), V (ventricular ectopic beat), F (fusion of a ventricular and a normal beat) and Q (pace, fusion of a pace and a normal beat, or beat cannot be classified). If the distance of the predicted QRS complex to the closest annotated beat (QRS complex) is less than ± 0.15 s, it is treated as the true positive (TP). Otherwise, it is considered as a false positive (FP), and the missed annotated beat is marked as a false negative (FN). The sensitivity (Sen), presion (Pre), error rate (Err), false negative rate (Fnr) of each type of beat, as well as the margin of TP to the closest annotated beat (mean ± std) were calculated to assess the performance, as shown by Eq. 1.

$$\begin{array}{cc} & \text{Sen}=\frac{\text{TP}}{\text{TP}+\text{FN}}\times 100\\ & \text{Pre}=\frac{\text{TP}}{\text{TP}+\text{FP}}\times 100\\ & \text{Err}=\frac{\text{FP}+\text{FN}}{\text{TP}+\text{FN}+\text{FP}}\times 100\\ & \text{Fnr}=\frac{\text{FN}}{\text{TP}+\text{FN}}\times 100 \end{array}$$ (1)

Table 2 summarizes the performance of the proposed method on the test datasets. The Sen, Pre, Err were 99.57%, 99.59% and 0.83% on AR_DS1, 99.88%, 99.63% and 0.48% on SV, 99.91%, 99.43% and 0.66% on ST-T, 99.94%, 99.47% and 0.59% on STC, 94.78%, 94.30% and 10.36% on NST, 98.33%, 99.93% and 1.74% on HIE, 96.10%, 94.35% and 9.13% on CPSC 2019. The proposed method achieved good results on the cross-dataset test. The Fnr for N type of beats was 0.34% on AR_DS1, 5.32% on NST, and less than 0.1% on the other datasets. For S type of beats, Fnr was approximately 0.1% on both AR_DS1 and SV, 2.4% on ST-T and 5.56% on NST. All S type of beats were successfully detected on STC. The Fnr for V type of beats was 1.56% on AR_DS1, 0.67% on SV, 2.33% on ST-T, 5.61% on STC, and 4.34% on NST. The datasets of CPSC2019 and HIE did not provide the annotations for beat types, and no F or Q type beats were found in STC and NST. The range of average distance between predicted and annotated heartbeat was from 21 milliseconds to 35 milliseconds, with a standard deviation of less than 25 milliseconds.

**TABLE 2:** The performance of proposed method.

Dataset	TP	FP	FN	Sen(%)	Pre(%)	Err (%)	Fnr (%)					Margin (milliseconds)
							N	S	V	F	Q	
AR_DS1	50803	208	218	99.57	99.59	0.83	0.34	0.10	1.56	0	0	23.03 ± 18.51
SV	184366	679	217	99.88	99.63	0.48	0.08	0.11	0.67	0	11.39	21.22 ± 16.50
ST-T	778424	4498	669	99.91	99.43	0.66	0.07	2.38	2.33	0.85	18.18	35.67 ± 23.18
STC	72855	387	46	99.94	99.47	0.59	0.04	0	5.61	-	-	31.54 ± 19.16
NST	24253	1466	1337	94.78	94.30	10.36	5.32	5.56	4.31	-	-	25.04 ± 20.58
CPSC2019	28299	1696	1148	96.10	94.35	9.13	-	-	-	-	-	31.31 ± 24.14
HIE	5596	4	95	98.33	99.93	1.74	-	-	-	-	-	34.33 ± 17.66

In order to compared with existing methods, the proposed method was retrained on the AR_DS1 and tested on the CPSC2019 dataset (with the tolerance 0.075 s), and retrained on the CPSC2019 and tested on NST datasets. The results were given on Table 3 and Table 4 respectively. On the CPSC 2019, compared with the state-of-the-art He’s method (He et al., 2021) the proposed method achieved a similar Sen (95.15% vs. 95.13%) and better Pre (91.75% vs. 82.03%) and Err (12.35% vs. 21.28%). On the NST, the performance of proposed detector was comparable to the best Cai’s method (Cai and Hu, 2020). The Sen of the proposed method was slightly better (95.59% vs. 95.55%), while the Pre and Err were slightly lower (91.03% vs. 92.93%, 12.64% vs. 10.92%, respectively).

**TABLE 3:** Comparison of the QRS complex detection performance on the CPSC 2019 (with of tolerance of 0.075s).

Method	TP	FP	FN	Sen(%)	Pre(%)	Err (%)
Ours (trained on AR_DS1)	28020	2521	1427	95.15	91.75	12.35
He (He et al., 2021)	28003	6140	1434	95.13	82.03	21.28
Hamilton (Hamilton, 2002)	25403	7350	4064	86.21	77.56	31.0
Pan (Pan and Tompkins, 1985)	24549	5939	4918	83.31	80.52	30.66

**TABLE 4:** Comparison of the QRS complex detection performance on the NST.

Method		Sen(%)	Pre(%)	Err (%)
Ours (trained on CPSC 2019)		95.59	91.03	12.64
Cai (Cai and Hu, 2020)	CNN	95.55	92.93	10.92
	CRNN	95.18	92.62	11.53
Khamis (Khamis et al., 2016)		86.21	77.56	69.0
Pan (Pan and Tompkins, 1985)		94.99	81.83	21.56

The ablation experiments were performed to evaluate the effectiveness of the improvements. A larger convolutional kernel size of 29 samples (MWLK) and a smaller convolutional kernel size of 9 samples (MWSK) were used to respectively replace the kernel size of 19 samples in the convolutional layers. And the model with single input channel (MWSC) was used to performed the detection on the first lead ECG-signal, similar to the work (Cai and Hu, 2020). In the training stage, the proposed model was trained with the border points of QRS complex (TWBP) and trained without simulated data (TWoSD) respectively. Additionally, it was reported that Fourier decomposition method based discrete Fourier transform (FDM-DFT) and discrete cosine transform (FDM-DCT) can effectively remove the baseline wander and power-line interference (Singhal et al., 2020), these two methods were adopted as alternatives for the butterworth filter. The Nemenyi test was applied to determine whether there were statistically significant differences in performance. The results were summarized in Tables 5–7.

**TABLE 5:** Result of Sen in ablation experiments.

Dataset	Algorithm	Overall	per record	*p* values of nemenyi test (with proposed method)
AR_DS1	Proposed	99.57	99.53 ± 1.56	-
	MWLK	99.78	99.82 ± 0.45	0.9
	MWSK	99.88	99.89 ± 0.23	0.9
	MWSC	99.81	99.82 ± 0.33	0.9
	TWBP	99.96	99.97 ± 0.09	0.67
	TWoSD	99.46	99.42 ± 1.91	0.9
	FDM-DCT	99.87	99.89 ± 0.26	0.9
	FDM-DFT	99.89	99.89 ± 0.26	0.9
SV	Proposed	99.88	99.90 ± 0.28	-
	MWLK	99.89	99.90 ± 0.23	0.9
	MWSK	99.96	99.97 ± 0.06	0.60
	MWSC	99.91	99.92 ± 0.22	0.82
	TWBP	99.95	99.96 ± 0.11	0.009
	TWoSD	99.81	99.87 ± 0.57	0.90
	FDM-DCT	99.91	99.93 ± 0.21	0.89
	FDM-DFT	99.93	99.94 ± 0.16	0.60
ST-T	Proposed	99.91	99.89 ± 0.37	-
	MWLK	99.96	99.95 ± 0.15	0.28
	MWSK	99.97	99.97 ± 0.08	0.13
	MWSC	99.77	99.77 ± 0.84	0.18
	TWBP	99.62	99.55 ± 1.47	0.004
	TWoSD	99.89	99.88 ± 0.26	0.76
	FDM-DCT	99.97	99.97 ± 0.07	0.016
	FDM-DFT	99.98	99.97 ± 0.06	0.074
STC	Proposed	99.94	99.89 ± 0.40	-
	MWLK	99.94	99.93 ± 0.13	0.9
	MWSK	99.99	99.98 ± 0.04	0.61
	MWSC	99.97	99.94 ± 0.18	0.9
	TWBP	99.97	99.97 ± 0.10	0.9
	TWoSD	99.82	99.83 ± 0.31	0.88
	FDM-DCT	99.93	99.92 ± 0.30	0.9
	FDM-DFT	99.95	99.94 ± 0.15	0.9
NST	Proposed	94.78	94.77 ± 8.98	-
	MWLK	96.66	96.63 ± 5.99	0.5
	MWSK	97.49	97.52 ± 4.66	0.22
	MWSC	96.63	96.67 ± 5.73	0.69
	TWBP	97.96	97.97 ± 4.24	0.05
	TWoSD	95.76	95.76 ± 6.82	0.9
	FDM-DCT	95.44	95.45 ± 7.75	0.9
	FDM-DFT	94.54	94.53 ± 9.83	0.9
CPSC2019	Proposed	96.10	96.51 ± 7.86	-
	MWLK	96.89	97.33 ± 6.77	0.097
	MWSK	97.89	98.12 ± 5.65	0.001
	MWSC	98.32	98.48 ± 4.73	0.001
	TWBP	97.47	98.13 ± 5.12	0.001
	TWoSD	93.54	95.11 ± 10.16	0.001
	FDM-DCT	95.54	96.03 ± 8.85	0.58
	FDM-DFT	96.68	97.08 ± 7.00	0.62
HIE	Proposed	98.33	98.43 ± 3.61	-
	MWLK	98.88	98.93 ± 3.24	0.32
	MWSK	98.96	99.00 ± 1.65	0.9
	MWSC	99.49	99.51 ± 1.04	0.045
	TWBP	98.21	98.33 ± 3.90	0.90
	TWoSD	86.98	87.75 ± 15.86	0.001
	FDM-DCT	98.88	98.92 ± 2.41	0.89
	FDM-DFT	98.95	98.99 ± 2.31	0.71

**TABLE 6:** Result of Pre in ablation experiments.

Dataset	Algorithm	Overall	per record	*p* values of nemenyi test (with proposed method)
AR_DS1	Proposed	99.59	99.51 ± 1.62	-
	MWLK	99.76	99.74 ± 0.57	0.9
	MWSK	99.23	99.16 ± 2.06	0.9
	MWSC	99.20	99.11 ± 3.28	0.9
	TWBP	98.25	98.23 ± 4.30	0.08
	TWoSD	99.57	99.46 ± 2.20	0.62
	FDM-DCT	99.49	99.43 ± 1.32	0.9
	FDM-DFT	99.76	99.73 ± 0.56	0.9
SV	Proposed	99.63	99.65 ± 1.90	-
	MWLK	99.91	99.90 ± 0.39	0.07
	MWSK	99.78	99.78 ± 0.58	0.9
	MWSC	99.52	99.55 ± 1.22	0.4
	TWBP	99.24	99.23 ± 2.18	0.001
	TWoSD	99.85	99.84 ± 0.54	0.15
	FDM-DCT	99.89	99.88 ± 0.29	0.54
	FDM-DFT	99.59	99.63 ± 2.41	0.03
ST-T	Proposed	99.43	99.28 ± 3.25	-
	MWLK	99.37	99.30 ± 4.71	0.06
	MWSK	98.91	98.77 ± 4.63	0.003
	MWSC	96.49	97.0 ± 8.18	0.12
	TWBP	98.39	98.26 ± 5.62	0.001
	TWoSD	99.29	99.13 ± 4.22	0.04
	FDM-DCT	98.93	98.82 ± 5.01	0.81
	FDM-DFT	99.50	99.41 ± 4.12	0.003
STC	Proposed	99.47	99.47 ± 2.06	-
	MWLK	99.50	99.51 ± 2.03	0.9
	MWSK	99.07	98.94 ± 2.63	0.001
	MWSC	99.30	99.16 ± 2.13	0.02
	TWBP	98.77	98.60 ± 2.44	0.001
	TWoSD	99.60	99.58 ± 1.57	0.9
	FDM-DCT	99.49	99.45 ± 1.86	0.9
	FDM-DFT	99.49	99.47 ± 1.97	0.9
NST	Proposed	94.30	94.09 ± 5.45	-
	MWLK	93.30	93.39 ± 7.79	0.9
	MWSK	89.87	90.30 ± 8.61	0.3
	MWSC	83.71	84.57 ± 10.94	0.001
	TWBP	89.60	90.30 ± 9.68	0.43
	TWoSD	92.60	92.60 ± 7.46	0.9
	FDM-DCT	93.83	93.72 ± 6.33	0.9
	FDM-DFT	95.78	95.51 ± 4.57	0.66
CPSC2019	Proposed	94.35	94.50 ± 11.47	-
	MWLK	94.63	94.80 ± 10.95	0.1
	MWSK	91.05	91.94 ± 13.96	0.001
	MWSC	89.56	91.14 ± 14.81	0.001
	TWBP	91.71	92.54 ± 12.21	0.001
	TWoSD	97.06	96.79 ± 7.97	0.001
	FDM-DCT	90.63	91.61 ± 14.38	0.001
	FDM-DFT	95.13	95.35 ± 10.22	0.11
HIE	Proposed	99.93	99.93 ± 0.45	-
	MWLK	99.98	99.98 ± 0.22	0.9
	MWSK	100.0	100.00 ± 0.0	0.9
	MWSC	99.93	99.93 ± 0.33	0.9
	TWBP	99.15	99.20 ± 1.97	0.22
	TWoSD	99.92	99.88 ± 0.64	0.9
	FDM-DCT	99.91	99.91 ± 0.66	0.9
	FDM-DFT	99.96	99.96 ± 0.25	0.9

**TABLE 7:** Result of Err in ablation experiments.

Dataset	Algorithm	Overall	per record	*p* values of nemenyi test (with proposed method)
AR_DS1	Proposed	0.83	0.92 ± 2.92	-
	MWLK	0.45	0.44 ± 0.77	0.9
	MWSK	0.89	0.95 ± 2.08	0.9
	MWSC	0.99	1.06 ± 3.39	0.9
	TWBP	1.79	1.80 ± 4.31	0.33
	TWoSD	0.96	1.05 ± 3.70	0.9
	FDM-DCT	0.63	0.67 ± 1.37	0.9
	FDM-DFT	0.36	0.37 ± 0.65	0.83
SV	Proposed	0.48	0.44 ± 1.91	-
	MWLK	0.21	0.20 ± 0.46	0.34
	MWSK	0.26	0.26 ± 0.60	0.9
	MWSC	0.57	0.53 ± 1.25	0.83
	TWBP	0.80	0.80 ± 2.18	0.001
	TWoSD	0.34	0.29 ± 0.78	0.58
	FDM-DCT	0.20	0.19 ± 0.36	0.21
	FDM-DFT	0.48	0.43 ± 2.42	0.007
ST-T	Proposed	0.66	0.82 ± 3.37	-
	MWLK	0.68	0.74 ± 4.73	0.04
	MWSK	1.12	1.26 ± 4.65	0.02
	MWSC	3.72	3.16 ± 8.38	0.08
	TWBP	1.97	2.12 ± 6.04	0.001
	TWoSD	0.82	0.99 ± 4.28	0.46
	FDM-DCT	1.10	1.20 ± 5.02	0.55
	FDM-DFT	0.52	0.62 ± 4.13	0.001
STC	Proposed	0.59	0.64 ± 2.12	-
	MWLK	0.56	0.56 ± 2.09	0.9
	MWSK	0.94	1.08 ± 2.64	0.02
	MWSC	0.73	0.89 ± 2.21	0.17
	TWBP	1.26	1.42 ± 2.48	0.001
	TWoSD	0.58	0.59 ± 1.74	0.9
	FDM-DCT	0.58	0.62 ± 2.09	0.9
	FDM-DFT	0.56	0.59 ± 2.00	0.9
NST	Proposed	10.36	9.99 ± 11.54	-
	MWLK	9.61	8.86 ± 11.48	0.3
	MWSK	12.17	11.27 ± 10.92	0.9
	MWSC	18.67	17.19 ± 13.22	0.004
	TWBP	12.04	10.93 ± 11.55	0.9
	TWoSD	11.04	10.33 ± 11.71	0.9
	FDM-DCT	10.19	9.62 ± 11.52	0.9
	FDM-DFT	9.25	8.97 ± 11.83	0.76
CPSC2019	Proposed	9.13	8.16 ± 13.35	-
	MWLK	8.16	7.29 ± 12.42	0.07
	MWSK	10.70	9.43 ± 14.57	0.11
	MWSC	11.79	9.99 ± 15.24	0.001
	TWBP	10.42	9.04 ± 12.82	0.001
	TWoSD	9.03	7.45 ± 12.22	0.9
	FDM-DCT	13.05	11.19 ± 15.98	0.001
	FDM-DFT	7.87	7.02 ± 12.06	0.008
HIE	Proposed	1.74	1.61 ± 3.85	-
	MWLK	1.14	1.08 ± 3.34	0.9
	MWSK	1.04	1.0 ± 1.65	0.9
	MWSC	0.58	0.56 ± 1.22	0.09
	TWBP	2.61	2.35 ± 4.98	0.9
	TWoSD	13.08	12.28 ± 15.91	0.001
	FDM-DCT	1.21	1.15 ± 2.81	0.9
	FDM-DFT	1.09	1.05 ± 2.30	0.79

## 5 Discussion

In this work, a simple and effective method was designed to detect the QRS complex on the ECG signal. A large-sized convolutional kernel was used in the convolutional layers, and the model was designed to have dual input channels. Furthermore, a novel training strategy was developed. The points on the border of the QRS complex were removed, and the artificially generated ECG segments with a high heart rate were included in the data augmentation. The proposed method achieved good results on the cross-dataset testing. The He’s method (He et al., 2021) and Cai’s method (Cai and Hu, 2020) are two state-of-the-art methods and have excellent performance. The He’s method adopted the U-Net combined with the Bidirectional LSTM modules. The Cai’s approach involves two models, one based on CNN and another based on CRNN. The CNN model composed of three parallel CNN blocks, a squeeze-and-excitation network (SENet) and three fully connected layers. The structure of the CRNN model was similar to the CNN model, with the addition of two LSTM layers before the SENet. Due to the use of sophisticated modules like LSTM and fully connected layers, these two methods have a large number of parameters (4,466,339 parameters in He’s method, 218,969 parameters and 2,708,417 parameters in Cai’s method). Compared with these state-of-the-art models, the proposed detector has fewer parameters and comparable results.

The QRS complex represents a short-duration electrical activity of the heartbeat and has clear differences from other waveforms. Due to the good ability of capturing the local feature, we thought that the CNN with a sufficiently sized receptive field can effectively learn the QRS complex features and identify the QRS complex, without the need for application of other sophisticated module. Consequently, the size of convolutional kernel becomes an essential issue in the for the FCN based model. The traditional CNN based algorithms with small-sized kernels can extract features that represent the detailed variations of QRS complex, which is advantageous to detect the QRS waves contaminated by noise. However, it increases the likelihood of misclassifying noise or other non-QRS waves with similar patterns as QRS complex, leading to more false positives. With the increased of kernel size, the model tend to overlook subtle variations in the QRS complex and instead focuses on a broader range of ECG waveforms. The model exhibits a tendency to enhance ECG waves that closely resemble the QRS complex, while effectively suppressing ECG waves with different shapes, such as QRS complexes contaminated by noise. As a result, the Pre increased and Sen decreased. In the experiments, compared to the MWSK, the MWLK has a lower Sen and a greater Pre, especially in the two dataset with relatively more number of noisy segments (CPSC2019 and NST). The Sen of MWLK and MWSK were 96.66% and 97.49% respectively on NST, 96.89% and 97.89% respectively on CPSC 2019, And the Pre of MWLK and MWSK were 94.63% and 91.05% in NST, 93.3% and 89.87% respectively in CPSC 2019. To achieve a balance between the Sen and Pre, an intermediate size of 19 samples was selected in the study, which has significantly lower Err on the ST-T and STC dataset, and no significant difference on other datasets.

Most of existing methods were designed to detect the QRS complex on the ECG signal with a single lead. Although some methods used dual input channels, the information of the second channel was extracted from the first channel. For instance, in the work (He et al., 2021), the ECG signal from the first channel was inverted and used as the second channel. In the work (Yuen et al., 2019), the gradient of the ECG signal from the first channel was used as the second channel. For the ECG signal with multiple input channels, we thought that when the noise was present on one channel of the multi-channel ECG signal, the DL-based detector can automatically utilize the information from other channels. This is advantageous in reducing the false positives. For the ECG signal with single-channel, as the signal of second channel was generated through signal replication, there would not be a significant increased in the Err, although the Sen and Pre may undergo some changes. The experimental results indicate that our method is helpful in improving performance. For the detection on the signals with two noisy dataset, although the Sen of MWSC was slightly higher (96.63% vs. 94.78% on NST, 98.32% vs. 96.10% on CPSC 2019), but the Pre (83.71% vs. 94.30% on NST, 89.56% vs.94.35% on CPSC 2019) and Err (18.67% vs. 10.36% on NST, 11.79% vs. 9.13% on CPSC 2019) were fundamental lower than proposed method.

In the training, the target of the DL model was generated by marking a binary label to the points on the ECG signal, and the label value was determined to the interval to the closest annotated QRS complexes. So, the different labels would be assigned to adjacent points with a similar presence on the border of QRS complex. It would confuse the model and consequently increase the number of false positives. Following the concept of CenterNet (Zhou et al., 2019), the QRS complex can be represented by a single point at the center of a bounding box, Hence, the points around the endpoints of the bounding box could be ignored in the training. It is reported that the duration of QRS comples is less than 150 milliseconds (Taylor, 2008), and the predicted QRS complex was considered as false positive if its distance to the closest annotated QRS complex is longer than 150 milliseconds. Consequently, the points whose interval to the closest annotated QRS complex from 75 milliseconds to 150 milliseconds can be removed in the training. Experimental results shows that TWBP had a lower Pre and higher Err compared to the proposed on all datasets. For the detection on the signals with two noisy dataset, although the Sen of MWSC was slightly higher (97.96% vs. 94.78% on NST, 97.47% vs. 96.10% on CPSC 2019), but the Pre (89.60% vs. 94.30% on NST, 91.71% vs.94.35% on CPSC 2019) and Err (12.04% vs. 10.36% on NST, 10.42% vs. 9.13% on CPSC 2019) were fundamental lower than proposed method.

During high-intensity exercise, the heart rate can reach a high level. However, most datasets contain only a small number of high heart rate segments. This may have a detrimental impact on the performance of the DNN model as it may miss many QRS complexes in the application. When the simulated segments were excluded from the training (TWoSD), the Sen reduced from 98.33% to 86.98%, and Err increased from 1.76% to 13.08% on the HIE. Experimental results show that the application of simulation data with a high heart rate is beneficial to train the model.

Noise attenuation plays a crucial role as a preprocessing step in QRS complex detection. While the noise suppression capability of the butterworth filter may not be as robust as Fourier-based methods, it can be effectively compensated for by utilizing a DNN model. This is attributed to the powerful representation capabilities of the DNN model, which enables it to overcome the limitations of the butterworth filter. The experimental results demonstrate that the proposed method maintains a comparable level with FDM-DCT and FDM-FFT on most datasets, without significant reduction.

The N, S and V are three primary types of beats, and the Fnr of S and V type of beats is lower than N type of beats. It can be attributed to several factors. Firstly, the rhythm of S and V type of beats deviates from the normal sinus rhythm and tends to be more unstable. Secondly, the morphology of S and V type of beats is more variable compared to N beats. Lastly, the volume of training data associated with S and V beats is substantially less than that of N beats. Therefore, it is more challenging to accurately detect S and V type of beats, resulting in a higher Fnr.

In the experiments, several hyperparameters and strategies were employed to prevent the overfitting. Firstly, the model was trained for a limited number of epochs, specifically 5 epochs, with early stopping based on validation error. This decision was made to strike a balance between allowing the model to learn meaningful patterns in the data and avoiding excessive training. Furthermore, a batch size of 32 was utilized during the training process. This choice of batch size ensures a sufficient number of samples are processed in each iteration, while simultaneously introducing more noise into the optimization process to prevent overfitting and avoid memorizing the training data. Additionally, a weight decay of 1e-8 was applied to the model’s parameters. This regularization technique encouraging the model to have smaller weights, resuling in preventing the model from becoming too complex.

The main limitation of proposed detector is the fixed threshold used for the determination of the QRS complex and without the search-back strategy. On the noisy segments, the response of the DNN model may be relative low. So many QRS complexes would be missed. It is generally thought the err-predicted is better than the miss detected (Cai et al., 2020). Therefore, an adapatively adjusted threshold with a search-back strategy can be applied, if no QRS complex was detected during a specific interval. Besides, the positional differences between predicted and annotated heartbeats were relatively large. In subsequent analyses, the impact of positional offsets can be mitigated by using a wider analysis window for heartbeat analysis, and by calculating the heart rate based on multiple heartbeat intervals.

## 6 Conclusion

In the paper, a DNN based algorithm with a novel training strategy was developed to detect the QRS complex on the ECG signal. After preprocessing the ECG signal, the DNN model was used to predict the probability of each point belonging to the QRS complex. Then local maximal points on the heat maps with the probability exceeding than the specified threshold were treated as the candidates. Finally, the NMS based post-preprocessing was performed to remove the false positives. The proposed method had a small number of parameters and achieved a good result on several public ECG datasets, indicating that it may be applicable to wearable ECG devices. The limitation of the work is the utilization of a fixed response threshold without the search back strategy. In the future work, we will try to address this limitation and increase the prediction heads of classification and delineation for the further analysis of heartbeats.

## Data Availability

Publicly available datasets were analyzed in this study. This data can be found here: https://physionet.org/about/database/#ecg.
